# Disgust and anxiety: What came first, the chicken or the egg?

**DOI:** 10.1192/j.eurpsy.2021.228

**Published:** 2021-08-13

**Authors:** M. Innocenti, G. Santarelli, V. Gironi, V. Faggi, L. Lucherini Angeletti, N. Giaquinta, V. Ricca

**Affiliations:** 1 Human Health Sciences, University of Florence, firenze, Italy; 2 Human Health Sciences, University of Florence, Firenze, Italy

## Abstract

**Introduction:**

Disgust is a basic emotion characterized by the feeling of revulsion and evoked by unpleasant stimuli such as contaminated food, poor hygiene and contact with sick or dead organisms. Disgust is a contributing factor to the development of several mental disorders including anxiety disorders (AD). Several studies have tried to explore the relationship between disgust and eating disorders (ED), with heterogeneous findings. Subjects with ED showed a heightened level of disgust sensitivity (DS) when compared with healthy controls (HC).

**Objectives:**

Our study aims to evaluate levels of disgust and anxiety in ED, AD and HC in order to assess associations between these two emotions.

**Methods:**

We enrolled 74 patients admitted to Psychiatric Unit of Careggi, 41 with diagnosis of Eating Disorder, 33 with Anxiety Disorders, and 40 healthy controls. We administered to all groups: Zung Anxiety Scale (ZSAS) and Disgust Propensity and Sensitivity Scale-revised (DPSS-r).

**Results:**

**Figure 1:**
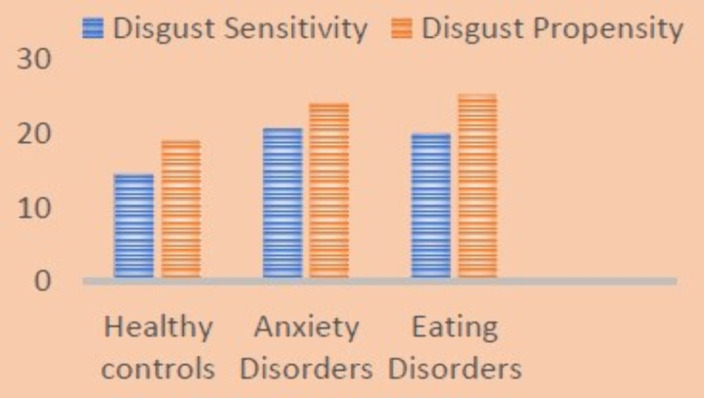

**Figure 2:**
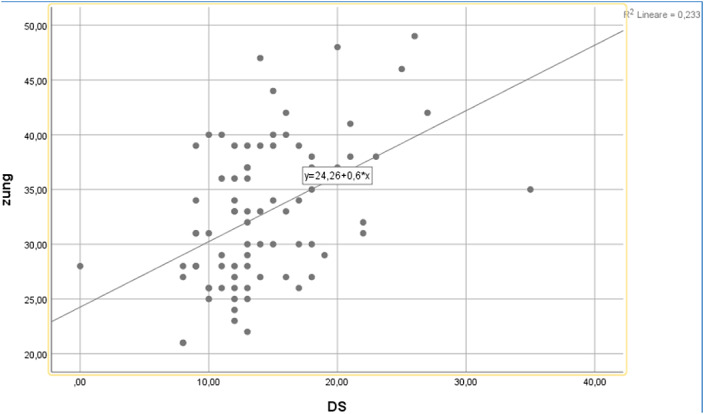

**Figure 3:**
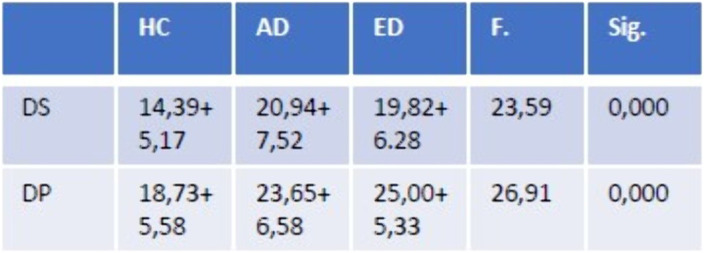

Both patients with anxiety disorders and eating disorders showed higher levels of disgust propensity and sensitivity than healthy controls. Moreover, there was no significant differences in anxiety, Disgust Propensity (DP) and Disgust Sensitivity levels between patients with eating disorders and anxiety disorders. Among healthy controls there was a significant association between DS and Anxiety levels (B: 0.579, T:3,416 p:0,001).

**Conclusions:**

Anxiety and disgust are typical emotions of anxiety disorders and eating disorders. However, they are increased both in anxiety and eating disorders and they are associated in healthy controls. The nature of this association needs to be deeply investigated.

**Disclosure:**

No significant relationships.

